# Accuracy of a magnetic resonance imaging‐based 3D printed stereotactic brain biopsy device in dogs

**DOI:** 10.1111/jvim.15739

**Published:** 2020-02-24

**Authors:** Sarah Gutmann, Dirk Winkler, Marcel Müller, Robert Möbius, Jean‐Pierre Fischer, Peter Böttcher, Ingmar Kiefer, Ronny Grunert, Thomas Flegel

**Affiliations:** ^1^ Department of Small Animal Medicine, Faculty of Veterinary Medicine University of Leipzig Leipzig Germany; ^2^ Department of Neurosurgery University Clinic of Leipzig, Faculty of Medicine Leipzig Germany; ^3^ Medical Engineering Fraunhofer Institute for Machine Tools and Forming Technology IWU Dresden Germany; ^4^ Department of Orthopedics, Trauma and Plastic Surgery University Clinic of Leipzig, Faculty of Medicine Leipzig Germany; ^5^ Small Animal Clinic, Department of Veterinary Medicine Free University of Berlin Berlin Germany

**Keywords:** 3D printing, brain biopsy, canine, MRI, neurosurgery, personalized

## Abstract

**Background:**

Brain biopsy of intracranial lesions is often necessary to determine specific therapy. The cost of the currently used stereotactic rigid frame and optical tracking systems for brain biopsy in dogs is often prohibitive or accuracy is not sufficient for all types of lesion.

**Objectives:**

To evaluate the application accuracy of an inexpensive magnetic resonance imaging‐based personalized, 3D printed brain biopsy device.

**Animals:**

Twenty‐two dog heads from cadavers were separated into 2 groups according to body weight (<15 kg, >20 kg).

**Methods:**

Experimental study. Two target points in each cadaver head were used (target point 1: caudate nucleus, target point 2: piriform lobe). Comparison between groups was performed using the independent Student's *t* test or the nonparametric Mann‐Whitney *U* Test.

**Results:**

The total median target point deviation was 0.83 mm (range 0.09‐2.76 mm). The separate median target point deviations for target points 1 and 2 in all dogs were 0.57 mm (range: 0.09‐1.25 mm) and 0.85 mm (range: 0.14‐2.76 mm), respectively.

**Conclusion and Clinical Importance:**

This magnetic resonance imaging‐based 3D printed stereotactic brain biopsy device achieved an application accuracy that was better than the accuracy of most brain biopsy systems that are currently used in veterinary medicine. The device can be applied to every size and shape of skull and allows precise positioning of brain biopsy needles in dogs.

**Abbreviations**
CT: computed tomography
MR: magnetic resonance
MRI: magnetic resonance imaging
STL: Standard Tessellation Language

## 1 INTRODUCTION

Most intracranial lesions in dogs can be reliably identified by computed tomography (CT) or magnetic resonance imaging (MRI). However, defining the specific underlying disease based only on advanced imaging can be challenging.^1^, ^2^, ^3^, ^4^, ^5^ In a study of brain lesions that were visible on MRI in dogs where the investigators only had to differentiate between gliomas and a presumptive cerebrovascular accident, there was a significant error in identifying the correct disease with 10% to 47% of cerebrovascular accidents are misinterpreted as gliomas.^6^ Conversely, up to 12% of gliomas are diagnosed as infarctions.^6^ Therefore, clinicians often are expected to make treatment recommendations based on presumptive, potentially erroneous diagnoses.

Histopathological examination of representative brain tissue specimens can increase diagnostic accuracy in those cases.^5^, ^7^, ^8^, ^9^, ^10^ However, the high complexity and vulnerability of the brain requires minimally invasive and highly precise techniques for obtaining brain biopsies. Stereotactic brain biopsy devices are often considered superior to an open surgical approach as they minimize injuries to the brain and surrounding tissues during the biopsy procedure.^11^ In the last 2 decades, several CT‐ and MRI‐guided stereotactic brain biopsy devices have been developed for veterinary use.^5^, ^7^, ^8^, ^9^, ^11^, ^12^, ^13^, ^14^, ^15^, ^16^, ^17^, ^18^, ^19^, ^20^, ^21^

The CT‐guided modified Pelorus Mark III stereotactic system was 1 of the first biopsy devices in veterinary medicine. The mean needle placement error is determined to be 3.5 mm (SD: 1.6 mm).^12^ In another study, a modified Laitinen frame system for CT‐guided stereotactic brain biopsy is used.^13^ The established needle placement error is 2.9 mm (SD: 1.08 mm). The mean needle placement errors of the Model 1430 MRI KOPF stereotactic system (David Kopf Instruments, Tojunga, CA) are reported with 0.9 mm (SD 0.9 mm) and 1.7 mm (SD 1.6 mm).^15^ The first MRI‐guided stereotactic neuronavigation system in veterinary use achieves a needle placement error of 1.79 mm (SD: 0.87 mm).^11^ Furthermore, a CT‐guided frameless stereotactic brain biopsy system shows a mean application accuracy of 2.9 and 4.3 mm.^16^ More recently, a targeting error of <3 mm is measured using another stereotactic MRI‐guided biopsy device^17^ and 1 study determines median needle placement errors of 1.55 mm (range: 1.1‐3.4 mm) and 1.5 mm (range: 0.9‐2.0 mm) using stereotactic headframes for brain biopsy.^19^ All of the aforementioned systems have 1 or more disadvantages: (1) Biopsy accuracy is not sufficient for all lesion types; (2) The system is so expensive that it might be cost prohibitive for routine veterinary use.

**Figure 1:**
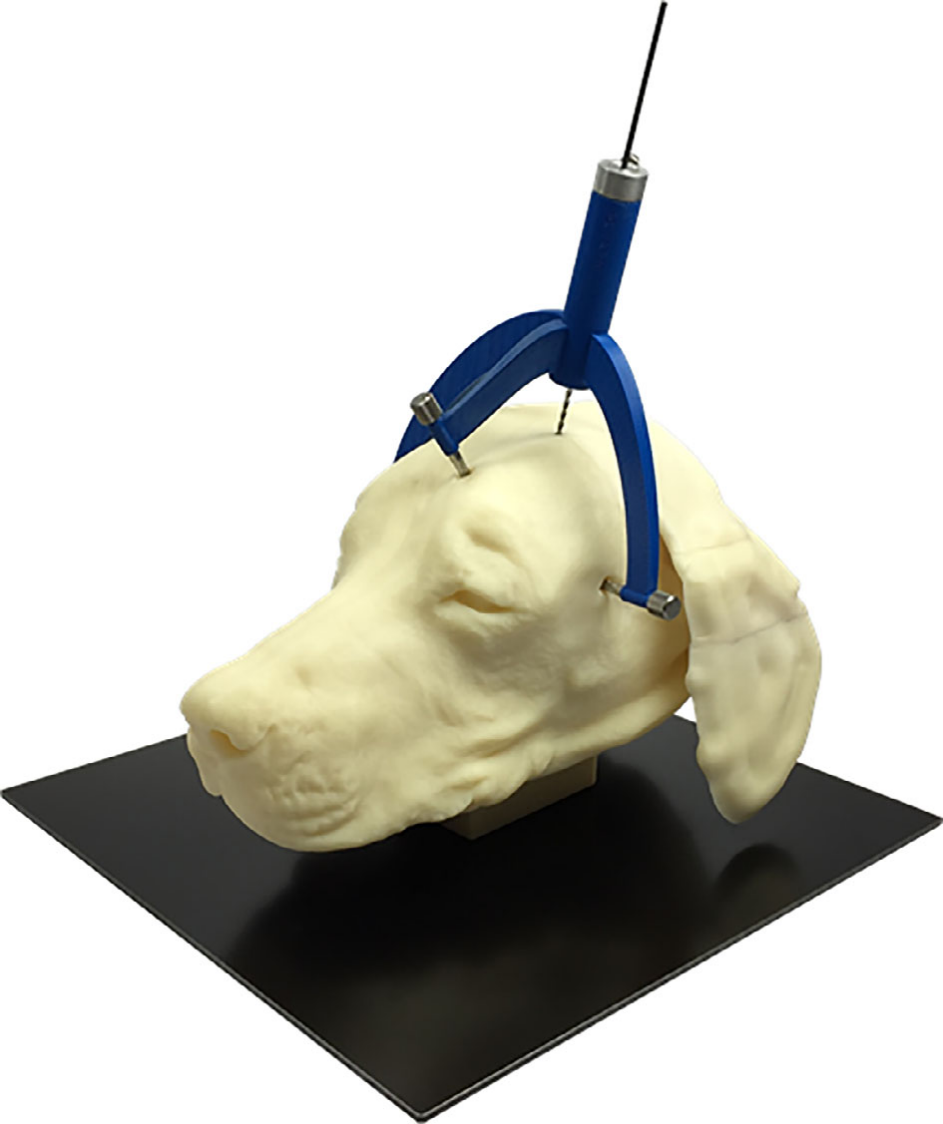
A dog model with a personalized, 3D printed stereotactic brain biopsy device connected to the head via bone anchors and screws with the biopsy needle in place

The aim of the study was to develop a new MRI‐based personalized, 3D printed stereotactic brain biopsy device for dogs that does not require expensive biopsy equipment and that has at least the same accuracy as currently available biopsy systems. This study presents the application accuracy of this new device (Figure 1) in targeting predefined intracranial points using a biopsy needle. Furthermore, the dog body weight and the depth of the target point were evaluated for any influence on procedural accuracy.

## 2 MATERIAL AND METHODS

The cadaver preparation, the computer‐aided manufacturing of the brain biopsy device, the brain biopsy needle placement, and the determination of the needle placement error were performed as described.^22^

## 3 CADAVER PREPARATION

Biopsy needle placement accuracy was tested in 22 canine cadavers. The cadavers were divided into small breed dogs (group 1) with a body weight of up to 15 kg and large breed dogs with a body weight of more than 20 kg (group 2). All dogs were euthanized for reasons unrelated to the study.

**Figure 2:**
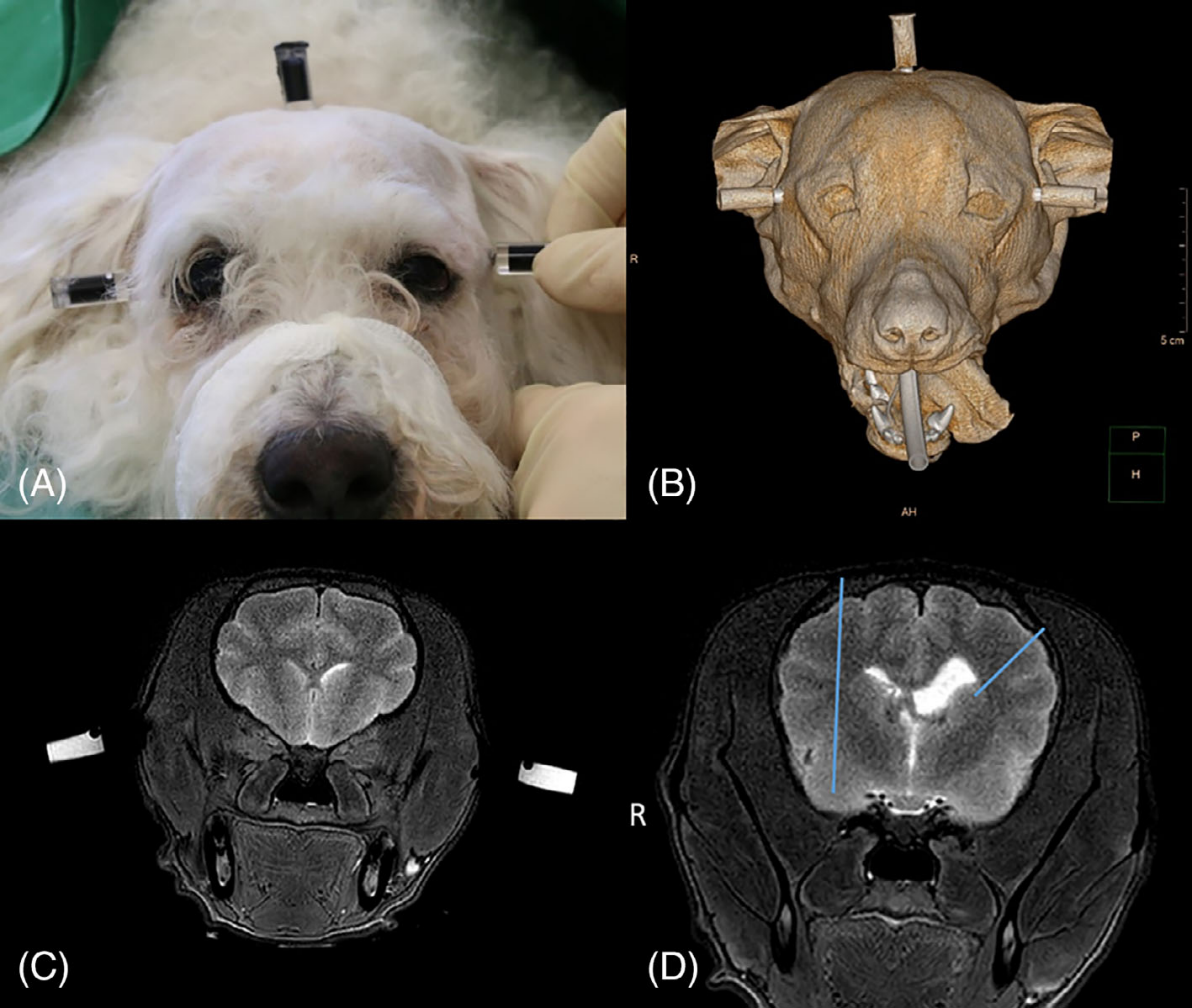
A, A canine head with 3 bone anchors and MRI/CT markers in place. Each side of the zygomatic arch and the occipital protuberance were used for fixation of the bone anchors. The MRI/CT markers were screwed to the inner thread of the bone anchors. The markers were small plastic cylinders filled with black‐stained diluted gadolinium. The MRI/CT markers attached to a canine head are visible in both examinations. B, CT surface reconstruction with the 3 attached markers (both sides of the zygomatic arch and on the occipital protuberance). C, Transverse T2‐weighted MR image of the brain with the attached markers on both sides of the zygomatic arch. D, A transverse T2‐weighted MR image of the head of a Havanese with predetermined biopsy needle trajectories (blue lines). On the left side, 1 point in the caudate nucleus and on the right side, another point in the piriform lobe were used as target points. The biopsy needle entered the brain surface in a gyrus, and it did not penetrate the ventricles

Each cadaver head was prepared as follows: Three specifically designed titanium bone anchors were screwed to predetermined bony points. These suitable points were located at the left and the right zygomatic arches and at the occipital protuberance. In very large dogs, the frontal bone overlying the frontal sinus was used instead of the occipital protuberance, as the latter was too solid for the attachment of the bone anchors without the need for predrilling. These bony points were determined because of the following conditions and tested in a pilot test on a cadaver dog: (1) The bone anchors should be grouped around the brain, so that the markers in the diagnostic imaging are nearly homogenously allocated; (2) The grouping around the brain was needed for a good and stable stand of the biopsy frame after attaching the frame to the bone anchors; and (3) The bony point should be superficially located, covered just by the skin, and they should be easy to palpate. The titanium bone anchors were small self‐cutting screws. The self‐cutting thread had a diameter of 2 mm and a length of 4 mm. The head of the bone anchors without markers extended 4.5 mm over the bony surface, allowing for skin closure over the markers in dogs that are alive. The contact face of the bone anchor to the bone of the head was 13.7 mm^2^. The head of the bone anchors had another internal thread allowing the attachment of either the CT/MRI markers (Figure 2A) or the biopsy frame.

Special markers that were visible on CT and MRI (Figure 2B,C) were screwed to the bone anchors. The markers were enclosed plastic cylinders with an outer diameter of 8 mm and a height of 18 mm. They were filled with diluted gadolinium (1 mL of gadolinium in 250 mL of physiological saline). The cylinder was fused to a screw that allows attachment of markers to the bone anchors. Following marker placement, CT‐ and MRI‐examinations of the entire head were performed with the cadavers in sternal recumbency. The scan parameters for CT‐ and MRI‐examinations are summarized in Table 1. The CT scan was only necessary to determine the accuracy of this biopsy device in the context of this study. The biopsy procedure in clinical cases will be solely based on MR images.

**Table 1:** CT and MRI examination devices and imaging parameters

	CT	MRI
Device	Philips Brilliance 8000 Mx	Ingenia
	6‐slice spiral CT	3‐Tesla MRI
	Philips Healthcare, Hamburg, Germany	Philips Healthcare, Hamburg, Germany
Setting	Head	Sequence: T2W gradient echo
	Solution: ultrahigh	Coil: knee^a^/head^b^
	Filter: bone	FOV: 140^a^/180^b^
	FOV: 140^a^/180^b^	TR: 3000
	ST: 0.7 mm^a^/1 mm^b^	TE: 90
	Collimation: 2 × 0.6^a^/6 × 0.75^b^	Matrix: 560
	Increment: −0.5	Voxel: isotropic (0.4 × 0.4 × 0.4)
		ST: 1 mm
		Gap: 0 mm

a Small dog group.

b Large dog group.

Abbreviations: FOV, field of view; ST, slice thickness; T2W, T2‐weighted sequence; TE, echo time; TR, repetition time.

Two target points were defined on transverse T2‐weighted MR images: 1 point in the left caudate nucleus and 1 point in the right piriform lobe. Those target points were points in the true geometrical sense of this term without any dimensions. In addition, an anticipated trajectory for each target point was drawn on the same MR image. The entry point at the brain surface was chosen in the middle of a gyrus. Care was taken to avoid penetration of the ventricles with any trajectory (Figure 2D).

## 4 COMPUTER‐AIDED MANUFACTURING OF THE BIOPSY DEVICE

**Figure 3:**
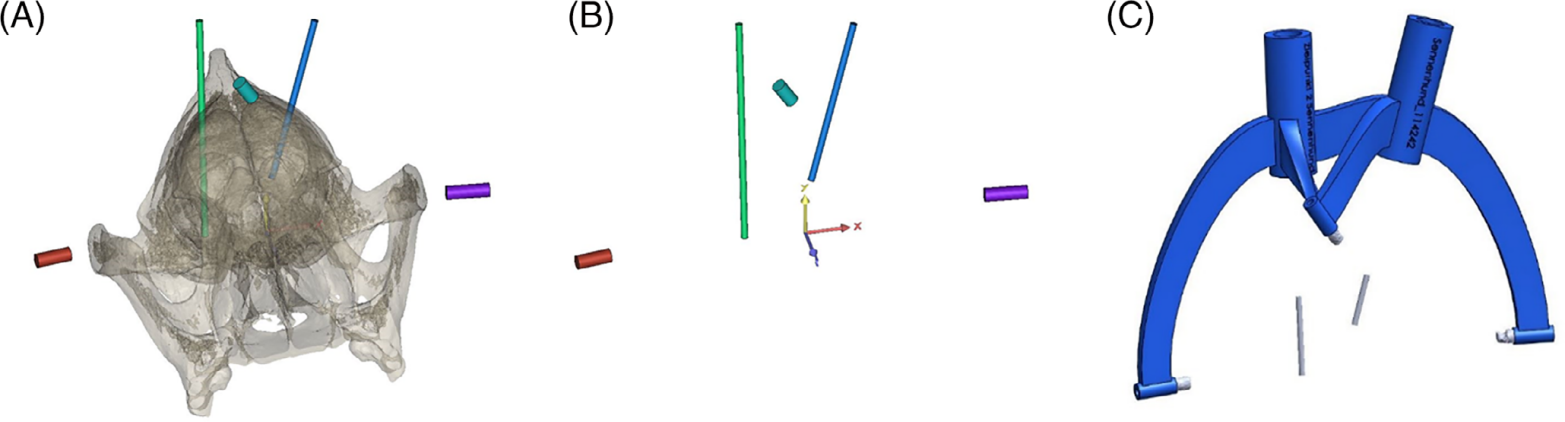
A 3D model of all 3 MRI/CT markers, the 2 target points and the 2 trajectories. After segmentation of the MRI data set, the markers and trajectories were idealized as cylinders with the help of the software program Mimics 16.0. A, Markers (red, violet, and cyan) and trajectories (green and blue) in relation to the canine skull. B, The 3 markers (red, violet, and cyan) and the 2 idealized trajectories (green and blue) in a 3‐dimensional coordinate system. C, The final design of the 3D frame using the software SolidWorks 2014. The frame (blue) consisted of 3 legs that were secured to bone anchors and 1 biopsy port for each trajectory for brain biopsy. Additionally, the 3 markers and the 2 trajectories (gray) are shown in relation to the frame

For the planning and construction of the biopsy device, the position of both target points and the planned trajectories were determined in relation to the 3 markers. To do so, MR images with markers, target points, and trajectories were imported into the program Mimics 16.0 (Mimics 16.0 [64bit], Materialize, Leuve, Belgium). Segmentation of the 3 markers and the anticipated trajectories was performed. A 3D model of all 3 markers, the 2 target points, and the 2 trajectories was generated. In that model, the markers and trajectories were represented by cylinders (Figure 3A,B). After Standard Tessellation Language (STL) exportation, all the objects were imported into the program 3‐matic (3matic [32bit], Materialize, Leuve, Belgium) by Materalise. The position and orientation of all the objects was manually checked, and the surface of all 5 cylinders was manually idealized. The coordinates of the distal and proximal surfaces of all the cylinders were determined and exported to the software MATLAB R2014a (MATLAB R2014a, Mathworks, Natick, Massachusetts) by Mathworks. With the help of the software, the coordinates were edited so that they could be used by the software SolidWorks 2014 (Solid Works 2014 [64bit], Dessault Systémes, Vélizy‐Villacoublay, France).

The final construction of the frame, consisting of 3 legs that were secured to the bone anchors and 1 biopsy port for each trajectory, was created with the software SolidWorks 2014 (Figure 3C). The prepared coordinates of the distal and proximal surfaces of all the cylinders were imported, and the geometry of the frame and the tool guide were adjusted. The final frame was exported as STL and sent to a 3D‐printer, Stratasys Fortus 900mc (Stratasys Fortus 900mc, Stratasys, Eden Prairie, Minnesota), that employs fused deposition modeling. The synthetic substance ABS M30 (ABS M30, Stratasys, Eden Prairie, Minnesota) was used for printing. The 3D‐printer worked with an accuracy of 0.1 mm in the x‐y plane and a slice thickness of 0.1778 mm.

## 5 BIOPSY NEEDLE PLACEMENT

**Figure 4:**
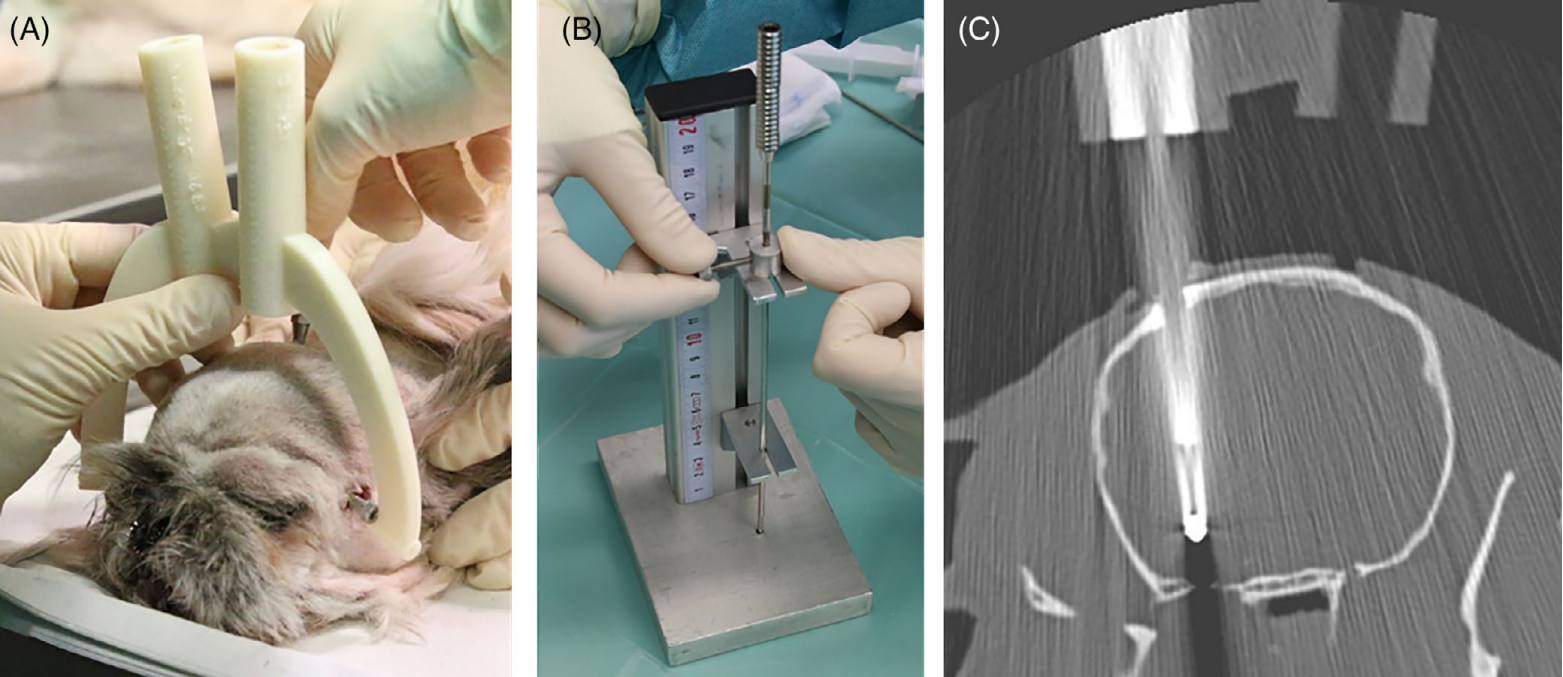
A, The personalized, 3D printed brain biopsy device was attached to the bone anchors with the help of specific screws. B, The depth of the biopsy needle was adjusted using a spacer with the help of a specific device. C, A transverse CT image with the biopsy needle in place (target point 2: piriform lobe)

The dog head was placed in sternal recumbency, and the individual frame was secured with its 3 legs to the 3 bone anchors using specifically designed screws (Figure 4A). Subsequently, minimally invasive access to the brain was obtained. A 1‐cm incision was made into the skin and the temporal fascia, and the temporalis muscle was bluntly separated until the skull surface was exposed. A round craniotomy opening of 3 mm in diameter was drilled (Electric Pen Drive, DePuy Synthes, West Chester, Pennsylvania) after placing a drill sleeve on the tool guide for the planned trajectory. The dura mater was perforated using a 0.3‐mm hypodermic needle. The drill sleeve was replaced by the biopsy needle sleeve, which was placed in the tool guide. A spacer was secured to the Sedan side‐cutting biopsy needle (Sedan side cutting needle, ELEKTA, Stockholm, Sweden; outer diameter of 2.5 mm) in order to restrict the needle advancement to the desired depth (Figure 4B). The biopsy needle was advanced through the tool guide with the needle sleeve into the brain parenchyma up to the maximum depth allowed by the spacer. A second CT scan of the skull with the biopsy needle in place was performed using the same scan parameters as in the first scan (Figure 4C). The entire procedure was repeated for the second target point.

## 6 DETERMINATION OF THE DEVIATION BETWEEN THE ANTICIPATED TARGET POINTS AND BIOPSY NEEDLE PLACEMENT

All 3 scans, the initial CT and MRI scans as well as the post‐biopsy CT scan, were fused. The fusion of the prebiopsy CT and MRI was achieved based on the markers that were visible in both modalities. The fusion of the pre‐ and post‐biopsy CT scans was performed based on the bony points. That way, all 3 scans could be imported into 1 coordinate system. The program 3‐matic was used for the fusion of all 3 examinations. After the fusion, the needle position was determined. The biopsy needle was idealized as a cylinder with a diameter of 2.5 mm. The coordinates of the needle tip were determined as the center point of the most distal part of the needle. The target point deviation in mm was defined as the deviation between the coordinates of the planned target point and the coordinates of the placed biopsy needle tip. Additionally, the free biopsy needle length from the lower end of the biopsy port to the needle tip was measured for each target point in each canine cadaver head.

## 7 STATISTICS

Statistical comparison of the data was performed using Microsoft Excel (Microsoft Excel, version 2013, Redmond, Washington) and SPSS software (SPSS software, version 24.0, IBM, Armonk, New York). The Kolmogorov‐Smirnov test was used to determine the normal distribution of the data. Data were presented as the mean with the SD or the median with the range based on their normality. The range was added in normally distributed data where it appeared to be appropriate. The comparison between the groups was performed using the independent Student's *t* test or the nonparametric Mann‐Whitney *U* Test. *P*‐values of .05 or less were considered statistically significant.

## 8 RESULTS

Twenty‐two canine cadaver heads were sampled. Eleven dogs were classified as small breed dogs with a body weight of less than 15 kg (group 1), whereas 11 dogs were classified as large breed dogs with a body weight of more than 20 kg (group 2). Group 1 contained the following breeds: Bichon Frise (n = 1), Chihuahua (n = 1), Dachshund (n = 1), French Bulldog (n = 1), Havanese (n = 1), Pug (n = 1), Coton de Tulear (n = 1), Shih Tzu (n = 2), and Yorkshire Terrier (n = 2). Group 2 included the following breeds: Bernese Mountain Dog (n = 2), English Bulldog (n = 1), German Shepherd (n = 1), husky (n = 1), Labrador Retriever (n = 3), and mixed breed dogs (n = 3). The mean body weight of the dogs in groups 1 and 2 were 7.6 kg (SD: 4.4 kg; range: 1.8‐14.8 kg) and 28.7 kg (SD: 5.9 kg; range: 22‐37.7 kg), respectively.

Two points were targeted in each cadaver head, resulting in a total of 44 target points. Twenty‐two points were located in the caudate nucleus (target point 1) and 22 points in the piriform lobe (target point 2). Technical problems were encountered in targeting 1 point in the caudate nucleus of 1 large breed dog and resulted in a target point deviation of 4.11 mm in this dog. This relevant deviation from the other values could be attributed to a technical‐related irregularity from the study protocol. In this dog (case number 21) no drill sleeve for drilling the minimal‐invasive access to the brain was used. So the craniotomy was made free‐handed because the drill sleeve was not present. In the reconstruction of the CT data set for the evaluation of the target point deviation of this case, contact of the biopsy needle with a bone edge of the craniotomy could be detected most likely caused by free hand drilling. This might have caused bending, so that the value of the target point 1 of the case number 21 was declared as a technical outlier and excluded from the results evaluations. Thus, the results of 43 target points (21 points located in the caudate nucleus and 22 in the piriform lobe) are reported. The median target point deviation for all the points in all the dogs was 0.83 mm with a range from 0.09 to 2.76 mm.

**Figure 5:**
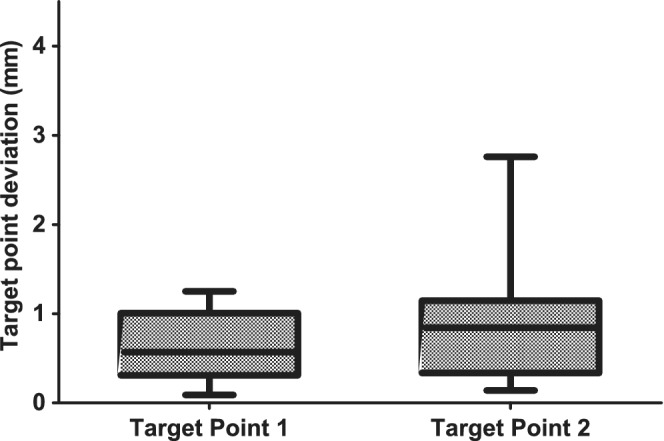
Box and whisker plots displaying the target point deviation of the 43 predetermined target points in mm using the MRI‐based personalized, 3D printed stereotactic brain biopsy device. The results are displayed for both target points separately (target point 1: caudate nucleus; target point 2: piriform lobe; whiskers represent minimum and maximum values)

The median target point deviation for all the target points in the caudate nucleus (target points 1, more superficial) and all the target points in the piriform lobe (target points 2, deeper) was 0.57 mm (range: 0.09‐1.25 mm) and 0.85 mm (range: 0.14‐2.76 mm), respectively (Figure 5). There was no significant difference in the target point deviation between the 2 points for all dogs taken together (*P* = 0.3).

The median target point deviation in the small breed dogs (<15 kg) for target point 1 was 0.5 mm (range 0.09‐1.00 mm) and for target point 2 was 0.84 mm (range 0.14‐2.08 mm). The mean free biopsy needle length for the small breed dogs in target points 1 and 2 was 46.62 mm (SD 7.08 mm) and 61.50 mm (SD 6.98 mm), respectively.

The median target point deviation in the large breed dogs (>20 kg) for target point 1 was 0.96 mm (range: 0.27‐1.25 mm) and for target point 2 was 0.93 mm (range: 0.29‐2.76 mm). The mean free biopsy needle length for the large breed dogs in target points 1 and 2 was 65.90 mm (SD 10.19 mm) and 78.84 mm (SD 7.81 mm), respectively.

There was no significant difference in the target point deviation between the 2 body weight groups for target point 2 in the piriform lobe (*P* = 0.36). However, the target point deviation for target point 1 in the caudate nucleus was significantly higher in group 2 (large breed dogs; median 0.96 mm (range: 0.27‐1.25 mm)) than in group 1 (small breed dogs; median 0.5 mm [range: 0.09‐1.00 mm]) (*P* < 0.05).

There was no linear correlation between the target point deviation and the free biopsy needle length (*R* ^2^ = 0.2067, Figure 6).

**Figure 6:**
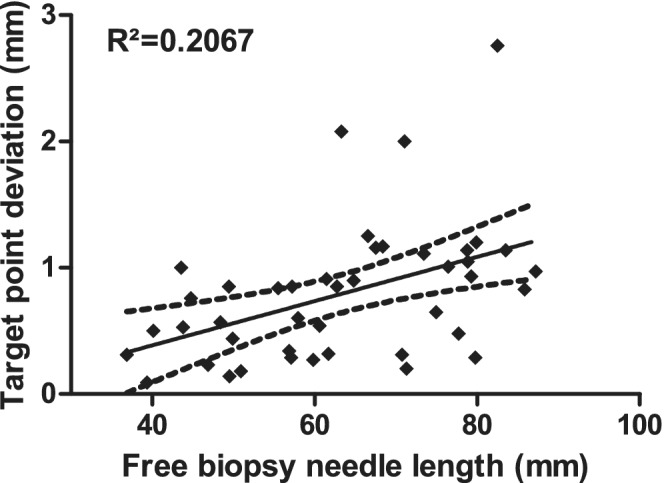
Comparison between the target point deviation and the free brain biopsy needle length for all 43 target points. Black line—best fit curve, broken lines—95% confidence interval

## 9 DISCUSSION

The MRI‐based personalized, 3D printed stereotactic brain biopsy device achieved excellent precision in targeting predefined intracranial points in the caudate nucleus and the piriform lobe. The overall median target point deviation was 0.83 mm (range: 0.09‐2.76 mm). The results are better than most of the previous studies of other stereotactic brain biopsy devices that are currently used in veterinary medicine, which have mean needle placement errors ranging from 0.9 mm to 4.3 mm 11, 12, 13, 15, 16, 17 and median needle placement errors of 1.5 mm and 1.55 mm.^19^ The error of system tested here is similar to the accuracies of stereotactic devices for brain surgery in human medicine.^23^, ^24^, ^25^, ^26^ The high application accuracy of the device presented here was achieved by rigid fixation of the biopsy device to the skull using bone anchors. Therefore, it does not allow any movement between the device and skull that can potentially occur when using a bite plate^11^, ^13^, ^14^, ^15^, ^16^, ^17^, ^19^, ^21^ or a face mask.^18^

In accordance with the results, the MRI‐based personalized, 3D printed stereotactic brain biopsy frame can be used to perform brain biopsies in dogs of varying weight and skull conformation. The smallest dog in this study was a Chihuahua with a dome‐shaped skull and a body weight of 1.8 kg. The largest dog was a dolichocephalic mixed breed dog with a weight of 38 kg. In contrast, some stereotactic brain biopsy devices have limitations with regard to the size of skulls they could be applied to,^15^ or the devices only were tested in a homogenous cohort of canine skulls.^11^, ^13^, ^14^, ^16^, ^20^ There are difficulties in applying other systems to small and dome‐shaped skulls.^12^ A bite plate system had problems in brachycephalic dogs caused by the specific round shape of their skulls and the malformations of the maxilla.^11^ A 3D printed patient‐specific facemask with an attached biopsy port can be used for brain biopsy,^18^ but might be problematic in brachycephalic dog breeds because of the use of the bridge of the nose and the nasal planum for the facemask. In the study presented here, 36% of dogs were brachycephalic breeds, such as French Bulldog, Pug, English Bulldog, or Shih Tzu breeds. In addition, some very small, brachycephalic dogs with dome‐shaped heads, such as Chihuahua or Yorkshire Terrier breeds, were included. It was easier to place the bone anchors and gain minimally invasive access to the brain in these dogs than in large normocephalic dogs as the thick masseter muscles in large breed dogs complicated the approach to the skull.

Figure 6 illustrates the relationship between free biopsy needle length (distance between the lower end of the biopsy port and the biopsy needle tip) and needle placement error. A previous study shows a negative correlation between body weight and needle placement error,^12^ but there is no significant relationship between the needle placement error and the target depth in another study.^11^ Two other separate studies interestingly conclude that more superficial lesions are biopsied less accurately than deeper lesions.^15^, ^16^ In the study presented here, there was no correlation between the free biopsy needle length and the target point deviation.

Our device can be used on the basis of MRI imaging, which is considered to be the gold standard for diagnosing intracranial lesions. Titanium bone anchors and markers filled with diluted gadolinium, which are visible in T1‐ and T2‐weighted MR images, facilitate MRI compatibility. Nevertheless, the markers are also visible on CT images. Therefore, the device could be used for CT‐based stereotactic brain biopsy or for intraoperative or postoperative CT examinations.

There are several advantages and a few disadvantages of the device presented here. It can be fixed to every skull size and shape as previously mentioned and the device can be rigidly fixed to the skull just using 3 screws without the need for fixing the skull to the table. This surgical setting allows more flexibility for the surgeon performing the procedure. Furthermore, the biopsy procedure is easy to perform, and it requires only 2 people. The surgeon does not need intensive training, like that needed for other biopsy systems, before using this stereotactic device.^12^, ^16^, ^20^, ^27^ However, the construction of the biopsy frame itself based on the MR images should be performed by a specifically trained person.

Additionally, the system allows the sampling of multiple brain specimens. The frame can either be constructed with multiple biopsy ports with different trajectories or, alternatively, a spacer secured to the biopsy needle can be used to vary the biopsy depth, allowing tissue sampling from different areas of the lesion along 1 trajectory.

One disadvantage of the system presented here is the separation of imaging and the biopsy procedure itself. Because the construction, 3D printing, and steam sterilization of the frame require up to 3 days, there are 2 procedures under general anesthesia that are necessary. Furthermore, the dog must have the bone anchors placed and covered by skin closed with 1 skin suture while the biopsy device is being constructed. Those bone anchors must stay in place until the biopsy has been performed. Therefore, there is a potential risk that the position of bone anchors may change or that they may loosen completely due to patient manipulation. This risk can be reduced by the application of a neck collar, but it cannot be eliminated completely. However, the bone anchors are screwed to the skull, and they protrude only 4.5 mm over the surface of the bone. Therefore, the risk of loosening should be low. Another limitation in use of the MRI‐based personalized, 3D printed stereotactic brain biopsy device is, that the trajectories that can be used for brain biopsy are fixed by the design. Therefore, the surgeon does not have the option to choose alternative trajectories if he/she encounters procedural technical problems or if the results of the smear preparation of the biopsy specimen indicate insufficient sampling. So the surgeon has to end the biopsy procedure with an alternative brain biopsy method (eg, image‐guided free‐handed brain biopsy).

Other potential sources of errors could be the accuracy of the 3D printer during the print, mistakes of the surgeon while attaching the markers or the frame to the bone anchors or while adjusting the needle depths, as well as mistakes made by the engineer while constructing the 3D biopsy frame on the PC.

There are some limitations of the study. The brain biopsy device is MRI‐based, but the target point deviation was determined with the help of CT examinations. Therefore, image fusion of both modalities was necessary to perform the study. Consequently, the determined needle placement error is the summation of the procedural error of the device itself and the error of the CT and MRI image fusion process. One might speculate that the study design might have artificially reduced the determined needle placement error and therefore improved the accuracy of the device. However, it is much more likely that the combination of 2 steps, each having intrinsic error, would have increased the total error of the procedure. Therefore, the needle placement error of the device presented here might even be lower than what was measured. Another limitation is the fact that the accuracy was tested for 2 localizations only.

In conclusion, the MRI‐based personalized, 3D printed stereotactic brain biopsy frame is a relatively inexpensive, highly precise, and economical way of sampling brain tissue in dogs of all sizes and with different head shapes.

## CONFLICT OF INTEREST DECLARATION

Authors declare no conflict of interest.

## OFF‐LABEL ANTIMICROBIAL DECLARATION

Authors declare no off‐label use of antimicrobials.

## INSTITUTIONAL ANIMAL CARE AND USE COMMITTEE (IACUC) OR OTHER APPROVAL DECLARATION

Authors declare no IACUC or other approval was needed.

## HUMAN ETHICS APPROVAL DECLARATION

Authors declare human ethics approval was not needed for this study.

## References

[1] Bohn AA , Wills TB , West CL , Tucker RL , Bagley RS . Cerebrospinal fluid analysis and magnetic resonance imaging in the diagnosis of neurologic disease in dogs: a retrospective study. Vet Clin Pathol. 2006;35:315‐320. 16967416 10.1111/j.1939-165x.2006.tb00138.x

[2] Cherubini GB , Mantis P , Martinez TA , Lamb CR , Cappello R . Utility of magnetic resonance imaging for distinguishing neoplastic from non‐neoplastic brain lesions in dogs and cats. Vet Radiol Ultrasound. 2005;46:384‐387. 16250394 10.1111/j.1740-8261.2005.00069.x

[3] Wisner ER , Dickinson PJ , Higgins RJ . Magnetic resonance imaging features of canine intracranial neoplasia. Vet Radiol Ultrasound. 2011;52:S52‐S61. 21392157 10.1111/j.1740-8261.2010.01785.x

[4] Wolff CA , Holmes SP , Young BD , et al. Magnetic resonance imaging for the differentiation of neoplastic, inflammatory, and cerebrovascular brain disease in dogs. J Vet Intern Med. 2012;26:589‐597. 22404482 10.1111/j.1939-1676.2012.00899.x

[5] LeCouteur RA . Current concepts in the diagnosis and treatment of brain tumours in dogs and cats. J Small Anim Pract. 1999;40:411‐416. 10516946 10.1111/j.1748-5827.1999.tb03113.x

[6] Cervera V , Mai W , Vite CH , Johnson V , Dayrell‐Hart B , Seiler GS . Comparative magnetic resonance imaging findings between gliomas and presumed cerebrovascular accidents in dogs. Vet Radiol Ultrasound. 2011;52:33‐40. 21322385

[7] Koblik PD , LeCouteur RA , Higgins RJ , et al. CT‐guided brain biopsy using a modified Pelorus Mark III stereotactic system: experience with 50 dogs. Vet Radiol Ultrasound. 1999;40:434‐440. 10528834 10.1111/j.1740-8261.1999.tb00371.x

[8] Moissonnier P , Blot S , Devauchelle P , et al. Stereotactic CT‐guided brain biopsy in the dog. J Small Anim Pract. 2002;43:115‐123. 11916055 10.1111/j.1748-5827.2002.tb00041.x

[9] Flegel T , Podell M , March PA , Chakeres DW . Use of a disposable real‐time CT stereotactic navigator device for minimally invasive dog brain biopsy through a mini‐burr hole. Am J Neuroradiol. 2002;23:1160‐1163. 12169475 PMC8185720

[10] Flegel T , Oevermann A , Oechtering G , Matiasek K . Diagnostic yield and adverse effects of MRI‐guided free‐hand brain biopsies through a mini‐burr hole in dogs with encephalitis. J Vet Intern Med. 2012;26:969‐976. 22708694 10.1111/j.1939-1676.2012.00961.x

[11] Chen AV , Wininger FA , Frey S , et al. Description and validation of a magnetic resonance imaging‐guided stereotactic brain biopsy device in the dog. Vet Radiol Ultrasound. 2012;53:150‐156. 22122485 10.1111/j.1740-8261.2011.01889.x

[12] Koblik PD , LeCouteur RA , Higgins RJ , et al. Modification and application of a Pelorus Mark III stereotactic system for CT‐guided brain biopsy in 50 dogs. Vet Radiol Ultrasound. 1999;40:424‐433. 10528833 10.1111/j.1740-8261.1999.tb00370.x

[13] Moissonnier P , Bordeau W , Delisle F , Devauchelle P . Accuracy testing of a new stereotactic CT‐guided brain biopsy device in the dog. Res Vet Sci. 2000;68:243‐247. 10877970 10.1053/rvsc.1999.0370

[14] Giroux A , Jones JC , Bøhn JH , et al. A new device for stereotactic CT‐guided biopsy of the canine brain: design, construction, and needle placement accuracy. Vet Radiol Ultrasound. 2002;43:229‐236. 12088316 10.1111/j.1740-8261.2002.tb00995.x

[15] Troxel MT , Vite CH . CT‐guided stereotactic brain biopsy using the Kopf stereotactic system. Vet Radiol Ultrasound. 2008;49:438‐443. 18833950 10.1111/j.1740-8261.2008.00403.x

[16] Taylor AR , Cohen ND , Fletcher S , Griffin JF , Levine JM . Application and machine accuracy of a new frameless computed tomography‐guided stereotactic brain biopsy system in dogs. Vet Radiol Ultrasound. 2013;54:332‐342. 23551960 10.1111/vru.12025

[17] Squires AD , Gao Y , Taylor SF , Kent M , Tse ZTH . A simple and inexpensive stereotactic guidance frame for MRI‐guided brain biopsy in canines. J Med Eng. 2014;2014:1‐7. 10.1155/2014/139535 PMC4782635 27006928

[18] James MD , Bova FJ , Rajon DA , Carrera‐Justiz S , Clemmons RM . Novel MRI and CT compatible stereotactic brain biopsy system in dogs using patient‐specific facemasks. J Small Anim Pract. 2017;58:615‐621. 28843044 10.1111/jsap.12705

[19] Rossmeisl JH , Andriani RT , Cecere TE , et al. Frame‐based stereotactic biopsy of canine brain masses: technique and clinical results in 26 cases. Front Vet Sci. 2015;2:20. 26664949 10.3389/fvets.2015.00020 PMC4672202

[20] Packer RA , Freeman LJ , Miller MA , Fauber AE , Morrison WB . Evaluation of minimally invasive excisional brain biopsy and intracranial brachytherapy catheter placement in dogs. Am J Vet Res. 2011;72:109‐121. 21194343 10.2460/ajvr.72.1.109

[21] Kani Y , Cecere TE , Lahmers K , et al. Diagnostic accuracy of stereotactic brain biopsy for intracranial neoplasia in dogs: comparison of biopsy, surgical resection, and necropsy specimens. J Vet Intern Med. 2019;33:1384‐1391. 30990928 10.1111/jvim.15500 PMC6524398

[22] Müller M , Winkler D , Möbius R , et al. A concept for a 3D‐printed patient‐specific stereotaxy platform for brain biopsy ‐ a canine cadaver study. Res Vet Sci. 2019;124:79‐84. 30856434 10.1016/j.rvsc.2019.02.007

[23] Widmann G , Eisner W , Kovacs P , et al. Accuracy and clinical use of a novel aiming device for frameless stereotactic brain biopsy. Minim Invas Neurosur. 2008;51:361‐369. 10.1055/s-0028-1085424 19061150

[24] Pezeshkian P , DeSalles AAF , Gorgulho A , et al. Accuracy of frame‐based stereotactic magnetic resonance imaging vs frame‐based stereotactic head computed tomography fused with recent magnetic resonance imaging for postimplantation deep brain stimulator lead localization. Neurosurgery. 2011;69:1299‐1306. 21725253 10.1227/NEU.0b013e31822b7069

[25] Lefranc M , Capel C , Pruvot AS , et al. The impact of the reference imaging modality, registration method and intraoperative flat‐panel computed tomography on the accuracy of the ROSA® stereotactic robot. Stereot Funct Neuros. 2014;92:242‐250. 10.1159/000362936 25170634

[26] Bradac O , Steklacova A , Nebrenska K , Vrana J , de Lacy P , Benes V . Accuracy of VarioGuide frameless stereotactic system against frame‐based stereotaxy: prospective, randomized, single‐center study. World Neurosurg. 2017;104:831‐840. 28454992 10.1016/j.wneu.2017.04.104

[27] Sidhu DS , Ruth JD , Lambert G , Rossmeisl JH . An easy to produce and economical three‐dimensional brain phantom for stereotactic computed tomographic‐guided brain biopsy training in the dog. Vet Surg. 2017;46:621‐630. 28462513 10.1111/vsu.12657

